# Multi-omics analysis of hexaploid triticale that show molecular responses to salt stress during seed germination

**DOI:** 10.3389/fpls.2024.1529961

**Published:** 2025-01-21

**Authors:** Dongxia Wang, Jiedong Li, Shiming Li, Jiongjie Fu, Baolong Liu, Dong Cao

**Affiliations:** ^1^ Department of Agriculture and Forestry, College of Agriculture and Animal Husbandry, Qinghai University, Xining, Qinghai, China; ^2^ Qinghai Province Key Laboratory of Crop Molecular Breeding, Northwest Institute of Plateau Biology, Chinese Academy of Sciences, Xining, Qinghai, China; ^3^ Key Laboratory of Adaptation and Evolution of Plateau Biota (AEPB), Northwest Institute of Plateau Biology, Chinese Academy of Sciences, Xining, Qinghai, China; ^4^ University of Chinese Academy of Sciences, Beijing, China

**Keywords:** hexaploid triticale, salt tolerance, germination stage, multi-omics, molecular response

## Abstract

The development of a salt-tolerant hexaploid triticale cultivar offers an economical and efficient solution for utilizing marginal land. Understanding how hexaploid triticales respond to salt stress is essential if this goal is to be achieved. A genome-wide association study (GWAS), along with transcriptome and proteome analyses, were used in the present study to determine the molecular responses to salt stress in hexaploid triticale. In total, 81 marker-trait associations for 10 salt-tolerance traits were identified in 153 hexaploid triticale accessions, explaining 0.71% to 56.98% of the phenotypic variation, and 54 GWAS-associated genes were uncovered. A total of 67, 88, and 688 differential expression genes were co-expressed at both the transcriptomic and proteomic levels after 4, 12, and 18 h of salt stress, respectively. Among these differentially expressed genes, six appeared in the coincident expression trends for both the transcriptomic and proteomic levels at the seed germination stage. A total of nine common KEGG pathways were enriched at both the transcriptomic and proteomic levels at 4, 12, and 18 h. After integrating GWAS-target genes with transcriptomics and proteomics approaches that the candidate gene *late embryogenesis abundant protein 14* (*LEA14*) was up-regulated at the transcriptomic and proteomic levels. *LEA14* contained important stress-responsive cis-acting regulatory elements that could be dynamically regulated by the binding of transcription factors (TFs). This suggested that *LEA14* was a key gene associated with salt tolerance in hexaploid triticale and could respond quickly to salt stress. This study improved understanding about the potential molecular mechanisms associated with hexaploid triticale salt tolerance and contributed to the breeding of salt-tolerant germplasms and the utilization of saline soils.

## 1 Introduction

Hexaploid triticale (×*Triticosecale* Wittmack, 2n = 6x = 42, AABBRR) is a man-made amphiploid grain-forage crop that is high yielding and resistant to abiotic stress (Blum, 2014). It is mainly produced in Poland, Germany, Belarus, France, Russia, and China and its planting area has continuously expanded with the increase in demand (FAOSTAT, 2017). Salt stress is an abiotic stress that seriously affects crop food or feed yield production and quality (Ondrasek et al., 2022). About 800 million hectares of global agricultural land is classified as low production soil due to salt salinity (F.A.O, 2008). In China, the land in the Qaidam Basin, Qinghai Province, is a typical agro-pastoral transition area and is suitable for forage based agriculture. However, the area shows widespread salinization. Soil salinization is mainly caused by irrigation and various ions, including sodium, potassium, and chloride (Haq et al., 2018; Jin et al., 2019), and sodium chloride (NaCl) is the most common salt component in the soil (He et al., 2022; Saleh et al., 2013). Soil salinization undesirably inhibits the growth and development of plants, including seed germination, root elongation, flowering, and fruiting (Deinlein et al., 2014), and breeding salt-tolerant cultivars is a highly effective and sustainable approach that enables the utilization of saline-alkali land in the Qaidam Basin and enhances hexaploid triticale production.

Seed germination is a key stage in successful crop production (Almansouri et al., 2001). The absorption of excess Na^+^ and Cl^−^ ions by plants can lower seed germination ability (Luan et al., 2014). Therefore, the genetic mechanisms associated with seed germination when plants respond to salt stress need to be clarified to develop new varieties with salt tolerance. Generally, salt tolerance is controlled by multiple genes and seed germination under saline conditions is influenced by various external and internal factors (Wahid et al., 2010). Previously, the seed germination loci and candidate genes controlling salt tolerance in model plants have been primarily identified by quantitative trait locus (QTL) analyses, RNA-seq approaches, and genome-wide association studies (Kong et al., 2021; Oyiga et al., 2018; Shi et al., 2017; Tu et al., 2021). The QTL mapping and genome-wide association study (GWAS) methods were used to identify genomic regions linked to salt tolerance traits in the Poaceae, such as relative fresh weight (RFW), relative germination potential (RGP), relative dry weight (RDW), relative shoot length (RSL), relative germination rate (RGR), and relative root length (RRL) (Batool et al., 2018; Ma et al., 2006; Park et al., 2011; Shi et al., 2017; Mwando et al., 2020). Some salt-responsive candidate genes have been identified in wheat and rice through GWAS approach (Liu et al., 2024; Javid et al., 2022; Oyiga et al., 2019; Quamruzzaman et al., 2022; Yu et al., 2023), such as *TATA modulatory factor* (Liu et al., 2024), *OsWRKY53* (Yu et al., 2023), *NRAMP-2* (Oyiga et al., 2019). The transcriptome and proteome are also used to elucidate changes and regulatory networks at the expression level, when responding to salt stress (Bahieldin et al., 2015; Yang et al., 2021; Yuan et al., 2018). The crop response to salt stress causes a series of signal transduction pathway changes, especially in pathways dependent on and independent of abscisic acid (ABA) (Yamaguchi-Shinozaki and Shinozaki, 2006). In the two signaling pathways, transcription factors (TFs) and their genes play vital roles in salt tolerance, for example: *AP2*/*ERF*, *WRKY*, *bHLH*, *WHIRLY*, *bZIP*, *MYB*, *DREB*, *MADS-box*, *GATA*, *NAC*, *WOX*, and *YABBY* (Hussain et al., 2021; Yoshida et al., 2014). Besides, many studies had reported that late embryogenesis abundant (LEA) proteins play a key role in various plants under salt stress (Jia et al., 2014; Park et al., 2011). A total of 51 LEA proteins had been identified and classified in *Arabidopsis* (Hundertmark and Hincha, 2008). Structural gene *LEA14*, as a member of LEA_2 subgroup, showed a positive correlation between the accumulation of LEA14 proteins and the salt tolerance (Jia et al., 2014). TFs, such as bHLH and WRKY, also regulated the expression level of LEA14 (Babitha et al., 2013). The aforementioned research has improved understanding about the molecular mechanisms associated with salt tolerance in hexaploid triticale.

Triticale is created through hybridization between wheat and rye and lacks a reference genome sequence. A genetic locus and the regulated pathways associated with important traits are not fully mined and elaborated in hexaploid triticales. Previous studies have only identified salt-tolerant cultivars and potential salt stress-responsive candidate genes in triticales based on physiology and transcriptome methods (Deng et al., 2020; Ramadan et al., 2023). Nevertheless, the molecular mechanism in triticales that responds to salt stress is poorly understood. The high quality reference genome sequences for wheat (RefSeq V2.1) and rye (Rabanus-Wallace et al., 2021) may provide references that could be used to identify the key genes in triticales, and have been successively applied to hexaploid triticales (Cao et al., 2022). In this study, loci and candidate genes associated with salt tolerance in hexaploid triticales were identified and molecular responses to salt stress during seed germination were revealed using transcriptome, proteome, and GWAS methods. This study provided reference information for gene fine-mapping and gene cloning and improved understanding about the molecular mechanism underlying salt tolerance in hexaploid triticales.

## 2 Materials and methods

### 2.1 Material and single nucleotide polymorphism markers

The plant materials containing hexaploid triticales accessions (n = 153) were provided by the Institute of Crop Sciences, Chinese Academy of Agricultural Sciences and the USDA-ARS National Small Grains Collection (http://www.ars-grin.gov/). Detailed information and the genotypes of these accessions have been published in Cao et al. (2022).

### 2.2 Salt tolerance evaluation

The intact seeds of 153 hexaploid triticales accessions were surface-sterilized using 1% NaClO for 10 min and then twice rinsed in sterile water. A set of 100 seeds was placed in a rectangular plastic box (125*55*125 mm) containing filter paper and 20 mL NaCl solution at a concentration of 200 mM was added to evaluate the salt tolerance of the accessions. The treatments, with three replicates per treatment, were either a distilled water (control) or 200 mM NaCl (salt treatment). The boxes were placed in an illuminated incubator at a constant temperature (conditions: 24 ± 2°C, 5,000 l× light intensity, 40% relative humidity, and a 16 h light/8 h dark cycle). The control and treatment solutions were replaced by distilled water or NaCl solution once a day, respectively. The number of germinated seeds, based on the emergence of a plumule, was counted each day. At the end of day 7, shoot length, shoot dry weight, root length, shoot fresh weight, root number, root fresh weight, and root dry weight were also measured according to previous methods (Javid et al., 2022). Several relative phenotype values were calculated for the GWAS (GP = (number of germinated seeds at three days/100) × 100% and GR = (number of germinated seeds at seven days/100) × 100%). The calculation of relative values for each characteristic (RGP, relative root dry weight (RRDW), RGR, RSL, relative root fresh weight (RRFW), RRL, relative root number (RRN), RFW, relative shoot fresh weight (RSFW), RDW, and relative shoot dry weight (RSDW) was based on the ratio of the measured value of the treatment to that of the control.

### 2.3 Genome-wide association study

The GWAS was carried out using three models by the Genome Association and Prediction Integrated Tool (GAPIT) in R package. The three models were the general linear model (GLM), fixed and random model circulating probability unification (FarmCPU), and the mixed linear model (MLM). A P-value threshold below 0.05/N indicated a significant link between SNPs and traits, with N referring to the total number of SNP markers applied in the association analysis (Yang et al., 2014). The valid SNPs were screened out by setting 1.27E−07 (−log10(p) = 6.90) as the significant P-value threshold.

### 2.4 Transcriptome profiling

The salt-tolerant variety QSM2 (ST) was identified based on the salt tolerance evaluation results and used in the transcriptome and proteome analyses. Each of the 100 QSM2 seeds was moistened with distilled water (control) or 200 mM NaCl (salt treatment) for 4, 12, and 18 h. Seed samples were collected at 4, 12, and 18 h, with three replications, and named STG4S, STG12S, and STG18S for the salt treatment, respectively, and STG4CK, STG12CK, and STG18CK for the control plants, respectively. All samples were immediately preserved at -80°C in liquid nitrogen. Next, a Tiangen RNAprep Pure Plant Kit (Tiangen Company, Beijing, China) was employed to isolate the total RNA. The quality of the total RNA obtained from each sample was determined according to Cao et al. (2019). A cDNA library (three replicates for each accession, 18 preparations in total) was constructed in accordance with the mRNA-Seq sample preparation protocol provided by the manufacturer (Illumina, Inc., San Diego, CA, USA). Then, Illumina paired-end sequencing technology and an Illumina HiSeq X instrument purchased from Novogene Co. Ltd. (Beijing, China) was used to sequence the cDNA library products with read lengths set at 150 bp. The detailed process and standards for the sequence data analysis are described in detail in Cao et al. (2019). We screened for significantly differentially expressed genes (DEGs) using |log_2_Ratio| absolute value ed genes a P-value threshold at a false discovery rate (FDR) ≤ 0.05.

### 2.5 Proteome profiling

Samples for protein extraction were prepared using the same method used for the RNA-seq. The protein preparation procedure and the Tandem mass tag (TMT)-based comparative peptidomics analyses are described in detail by Ma et al. (2021). In brief, lysis buffer [150 mM Tris-HCl pH 8.0 + 100 mM DTT + 4% (w/v) SDS] was used to extract the total protein from seeds using a three step process: 1) seeds were disrupted in a homogenizer by agitation and then boiled for 5 min; 2) the samples underwent further ultrasonication and then boiled again for 5 min; and 3) 15 min centrifugation at 16,000 rpm was performed to remove undissolved cellular debris. A BCA Protein Assay Kit (Bio-Rad, Hercules, CA, USA) was used to quantify the collected supernatant. The protein digestion was based on the filter-aided sample preparation procedure, as presented by the method of Wiśniewski et al. (2009). Finally, TMT reagents were added to label the obtained peptides as per the instructions of the manufacturer (Thermo Fisher Scientific, Scotts Valley, CA, USA). An Agilent 1290 HPLC (Agilent, Santa Clara, CA, USA) operated at 0.3 mL/min and equipped with a Waters XBridge BEH130 column (C18, 3.5 μm, 2.1 × 150 mm) (Waters, City, state, USA) was used to fractionate the TMT-labeled peptide mixture. A total of 30 fractions were obtained from each peptide mixture, which were subsequently concatenated to 15. Later, a nano LC-MS/MS analysis was carried out on the dried fractions.

An Easy nLC (Thermo Fisher Scientific)-coupled Q Exactive mass spectrometer was used to perform the LC-MS analysis. The acquired raw files from the LC-MS/MS were uploaded to Proteome Discoverer 2.4 software (version 1.6.0.16) to interpret the data and identify proteins against the database. A cutoff ratio fold-change of >1.20 or < 0.83 together with P-values < 0.05 were used to screen significant differentially expressed proteins (DEPs). The UniProtKB/Swiss-Prot, Gene Ontology (GO), and Kyoto Encyclopedia of Genes and Genomes (KEGG) databases were employed to extract information for sequence annotation. The enriched GO and KEGG pathways were identified using a nominally statistical significance of p < 0.05.

### 2.6 Promoter cis-element analysis

The wheat genome project (RefSeq V2.1) database was used to acquire promoter sequences (2 kb upstream of the start site of translation) for all late embryogenesis abundant protein (LEA) genes. The PLACE database (http://www.dna.affrc.go.jp/PLACE/signalscan.html) was applied to the *LEA* gene promoters to predict the transcriptional response elements (Higo et al., 1999).

### 2.7 Quantitative real-time PCR analysis

The transcriptome results were verified by means of a qRT-PCR based on the biological replication of RNA used in the RNA-seq procedure. **Supplementary Table S1** presents the primers for six genes that responded to salt stress. Then qRT-PCR validation was performed using ChamQUniversal SYBRqPCR Master Mix (Vazyme, Nanjing, China) following the manufacturer’s specifications. The reaction was executed using the following mixture: 2 × ChamQ Universal SYBRqPCR Master Mix (10 µL), forward and reverse primers (0.4 mL), cDNA (1 µL), and ddH_2_O (8.2 µL). The reaction mixture procedure was pre-denaturation for 30 s at 95°C; 40 cycles of 5-s cyclic reaction at 95°C and 54°C and 54cles and finally a melt curve procedure that went from 15 s at 95°C to 60 s at 60°C, and then 15 s at 95°C. The gene-specific RT-PCR products underwent amount normalization using the wheat actin gene as a control. The 2^−ΔΔ^ Ct method was used to calculate relative expression with three biological replicates.

### 2.8 Bioinformatics analysis

Microsoft Excel 2016 (Microsoft, Redmond, WA, USA) plus SPSS 11.5 software(SPSS Inc, Chicago, Ill, USA) were used to analyze the phenotypic data for all traits. Box and violin plots for phenotypic values were drawn using R software with the ggplot2 package. The R software package “corrplot” was utilized to obtain Pearson’s correlation coefficients for salt treatment trait pairs.

## 3 Results

### 3.1 Phenotypic variation and evaluation of salt tolerance in hexaploid triticale

**Figure 1:**
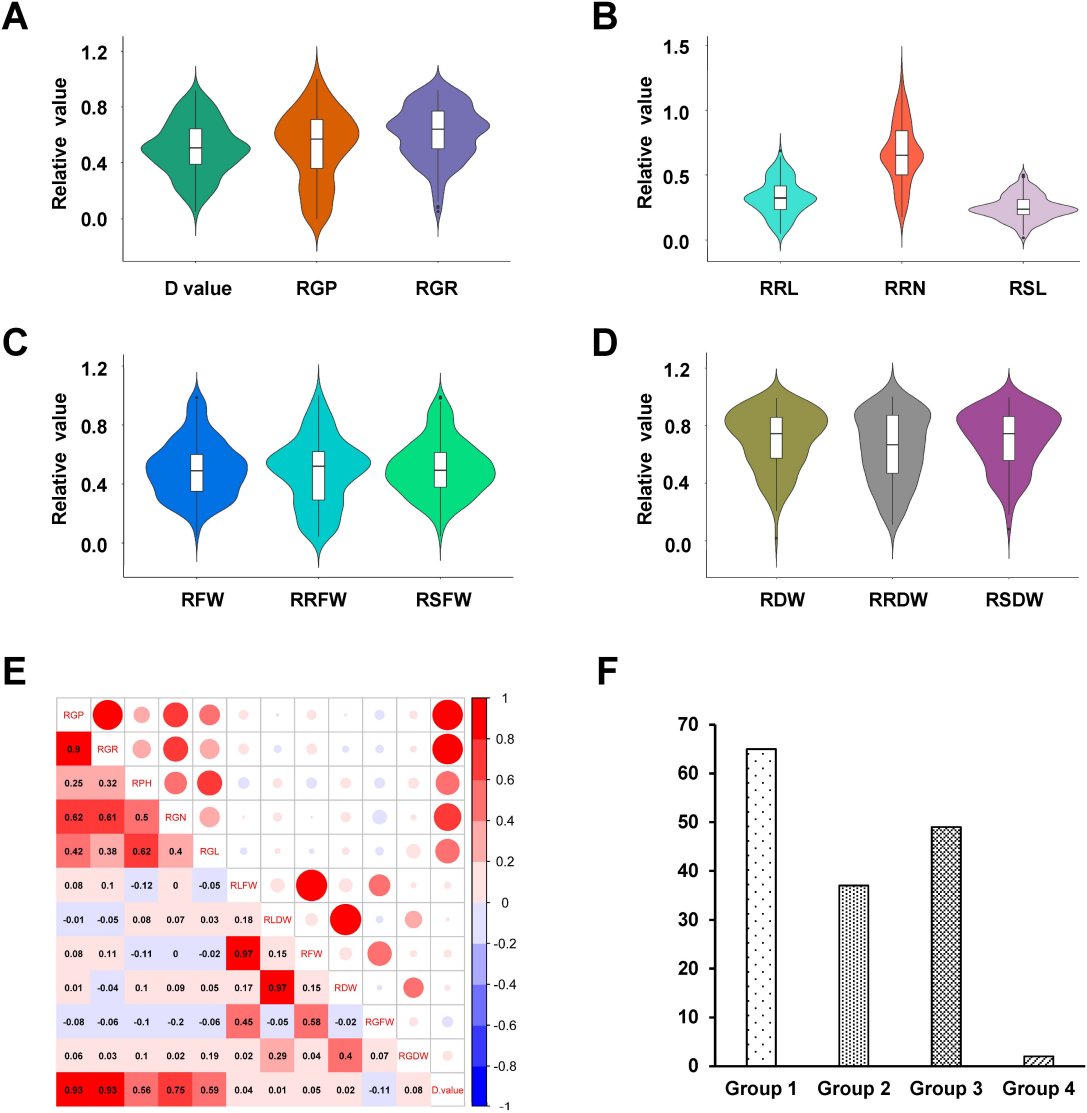
Phenotypic variation in salt tolerance among 153 hexaploid triticale accessions. **(A–D)** Violin plot of phenotypic indexes: **(A)** D value, relative germination potential (RGP), and relative germination rate (RGR); **(B)** relative root length (RRL), relative root number (RRN), and relative shoot length (RSL); **(C)** relative fresh weight (RFW), relative root fresh weight (RRFW), and relative shoot fresh weight (RSFW); and **(D)** relative dry weight (RDW), relative root dry weight (RRDW), and relative shoot dry weight (RSDW). **(E)** Correlations among all phenotypic indexes. **(F)** Hexaploid triticale groups based on D value.

The salt tolerance of 153 hexaploid triticale accessions was evaluated under 200 mM NaCl stress at the germination stage. Salt stress significantly inhibited all indexes compared to normal conditions (**Supplementary Figure S1**). The relative values for all the traits associated with salt stress were calculated and used to assess the 153 hexaploid triticale accessions for salt tolerance. The phenotypic distribution appeared continuous, variability was high (**Figures 1A–D**), and the trait pairs exhibited significant positive correlations (**Figure 1E**). The D value of the weighted membership function was used to comprehensively evaluate salt tolerance and the 153 hexaploid accessions had a D value that varied within the range 0.05–0.92 (**Figure 1A**). These 153 hexaploid triticale accessions were sorted and clustered according to their D value and divided into four groups (**Figure 1F**). Among these accessions, the highest and lowest D values were 0.92 (QSM2, clade I) and 0.05 (PI429196, clade IV), which indicated that these two accessions were the most salt-tolerant and salt-sensitive, respectively. A phenotype observation at the germination stage was undertaken using these two varieties and they were also used to identify the genes and proteins responding to salt stress.

Phenotypic variation in salt tolerance among 153 hexaploid triticale accessions. (A–D) Violin plot of phenotypic indexes: (A) D value, relative germination potential (RGP), and relative germination rate (RGR); (B) relative root length (RRL), relative root number (RRN), and relative shoot length (RSL); (C) relative fresh weight (RFW), relative root fresh weight (RRFW), and relative shoot fresh weight (RSFW); and (D) relative dry weight (RDW), relative root dry weight (RRDW), and relative shoot dry weight (RSDW). (E) Correlations among all phenotypic indexes. (F) Hexaploid triticale groups based on D value.

### 3.2 GWAS of salt tolerance in hexaploid triticale

Loci controlling complex traits can be identified in natural populations using a GWAS approach. To reveal the genetic variations in the salt tolerance shown by hexaploid triticale at the germination stage, SNPs and 12 traits with salt tolerance were analyzed using a GWAS. We used three models to detect 81 marker-trait associations (MTAs) related to salt tolerance traits (**Supplementary Table S2**). The number of MTAs for RGP, RGR, RRN, RRL, RSL, RRFW, RFW, RSDW, RDW, and the D values were 17, 36, 9, 3, 2, 1, 12, 12, 14, and 28, respectively. These MTAs were located on 15 chromosomes, except for 2R, 3R, 6A, 6R, 7A, and 7R, and accounted for 0.71–56.98% of the phenotypic variation. Among them, 31 belonged to pleiotropic MTAs controlling two traits (**Supplementary Table S2**). Three MTAs (3A_486188954, 5A_446387270, and 5A_614259381) were located synchronously by the three models, which indicated they were stable and reliable loci linked to RGR, RFW, RSDW, and RDW (**Supplementary Table S2**).

**Figure 2:**
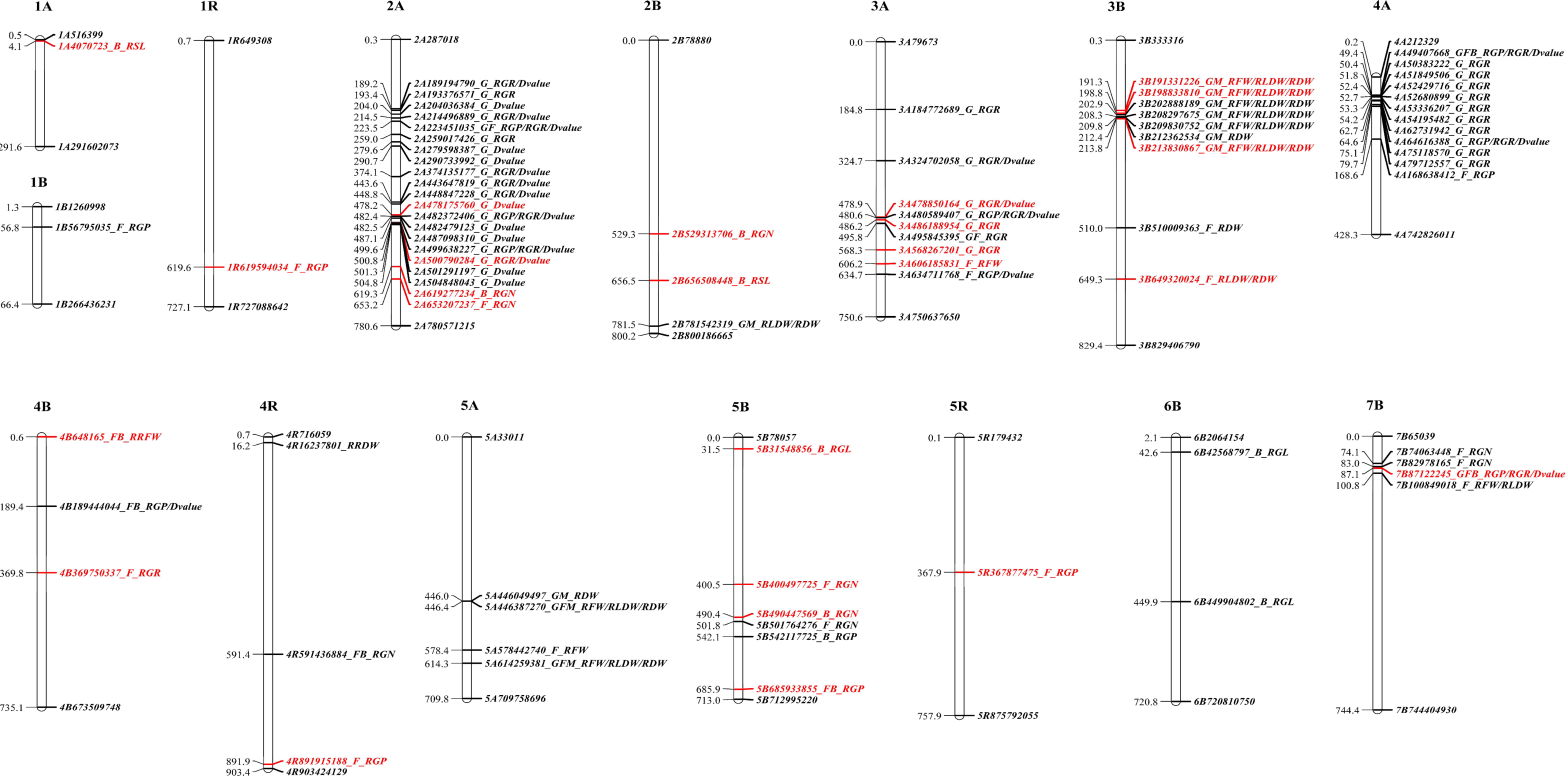
Physical maps of 26 significant marker-trait associations.

High quality references involving wheat and rye serve as advantageous and useful resources for determining the candidate genes constituting marker loci associated with salt tolerance in hexaploid triticale. Based on the linkage disequilibrium (LD) distance for the 153 hexaploid triticale (Cao et al., 2022), the wheat and rye genomes were predicted using 54 genes that were related to 26 significant MTAs (**Supplementary Table S3**; **Figure 2**). These genes were annotated using protein databases (**Supplementary Table S3**). According to the functional annotations and previous reports, the functions of partial candidate genes identified by this GWAS analysis were verified as playing key roles in resisting salt stress in other plants, such as *wall-associated receptor kinase* (Hou et al., 2005), *calcium-dependent kinase* (Asano et al., 2012; Mahajan et al., 2008), *serine/threonine-protein phosphatase* (Chen et al., 2014; Liu et al., 2000; Liao et al., 2016), *phospholipase D* (Bargmann and Munnik, 2006; Ji et al., 2017; Vadovič et al., 2019), *polyol transporter* (Saddhe et al., 2021), *dirigent protein* (Wang et al., 2022), *late embryogenesis abundant protein* (Jia et al., 2014; Park et al., 2011), *G-type lectin S-receptor-like serine/threonine-protein kinase* (Sun et al., 2013), *isocitrate lyase* (Yuenyong et al., 2019), *histone H2B* (Chen et al., 2019; Zhou et al., 2017), *bZIP* (Schütze et al., 2008; Zhang et al., 2008), and *WRKY* (Ali et al., 2018; Wang et al., 2018). Notably, the reliable MTA 3A_486188954 predicted *LEA14*, which responds positively to salt stress (Jia et al., 2014; Park et al., 2011). No candidate genes were predicted by the other two reliable MTAs. This further verified the authenticity of the MTAs identified by the GWAS results.

### 3.3 Global analysis of salt-related genes in hexaploid triticale

**Figure 3:**
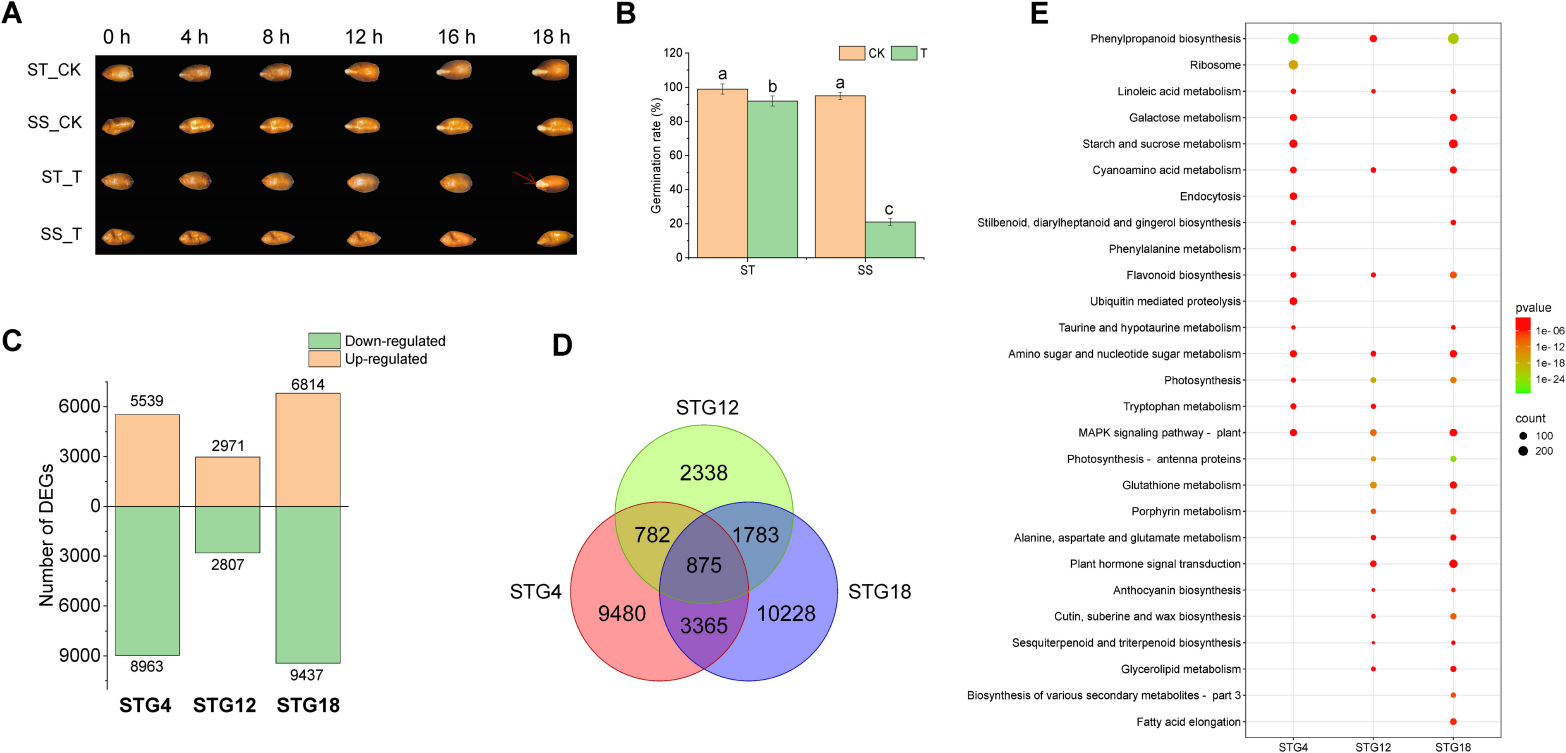
Phenotypic diversity and transcriptome analysis results under salt stress at the germination stage. **(A)** Seed morphology of salt-tolerant variety QSM2 (ST) and salt-sensitive variety PI429196 (SS) under normal and salt stress conditions. **(B)** Difference in germination rate (GR) between QSM2 (ST) and PI429196 (SS) under normal and salt stress conditions. **(C)** Number of DEGs in ST under normal and salt stress conditions at 4, 12, and 18 h. **(D)** Venn diagram of DEGs in ST under normal and salt stress conditions at 4, 12, and 18 h. **(E)** Enriched KEGG pathways for DEGs in ST under normal and salt stress conditions at 4, 12, and 18 h. Different letters in columns indicate statistically significant differences (p < 0.05).

The germination processes for salt-tolerant variety QSM2 (ST) and salt-sensitive variety PI429196 (SS) were observed under normal and salt stress conditions. Notably, the salt-tolerant variety QSM2 showed complete germination at 18 h under salt stress, whereas PI429196 was inhibited (**Figure 3A**). QSM2 displayed a remarkably raised germination rate compared to PI429196 (**Figure 3B**). The gene expression dynamics at the germination stage of salt-resistant variety QSM2 were characterized by RNA-seq using seeds incubated for three periods of time (4, 12, and 18 h) under normal or salt stress conditions. In total, 18 libraries (six samples, each of which had three biological replicates) were prepared for sequencing. Nearly 123 Gb clean data for all samples were obtained (**Supplementary Table S4**). The comparison of DEGs with the same incubation times under normal and salt conditions yielded three different comparison groups: STG4S_vs_STG4CK, STG12S_vs_STG12CK, and STG18S_vs_ STG18CK. A total of 14,502, 5,778, and 16,251 DEGs were identified in the three different comparison groups, respectively (**Figure 3C**). A total of 875 genes were shared by the three comparison groups (**Figure 3D**). The combined GWAS results indicated that 12 candidate genes showed differential expression levels at least one time point (**Supplementary Table S3**). Notably, only one gene, late embryogenesis abundant protein 14 (LEA14), had up-regulated expression at all three time points (**Supplementary Table S3**).

The different time points displayed divergent KEGG pathways (**Figure 3E**; **Supplementary Table S5**). Five KEGG pathways were shared by the three comparisons: cyano-amino acid metabolism, flavonoid biosynthesis, amino sugar and nucleotide sugar metabolism, linoleic acid metabolism, and phenylpropanoid biosynthesis. As the salt stress time increased, more up-regulated genes were found in plant MAPK signaling pathways. The functional categories of DEGs during salt stress were annotated using GO functional classification. After 4, 12, and 18 h salt stress, 719, 771, and 1,022 GO terms were significantly enriched in QSM2, respectively (**Supplementary Table S6**). More DEGs in the salt stress response group were enriched in the Biological Process category (**Supplementary Table S6**). The above findings suggested that triticale mostly used the same terms and pathways and that salt stress triggered a basic salt response, such as MAPK signaling and ABA response pathways.

### 3.4 Global analysis of salt-related proteins in triticale

**Figure 4:**
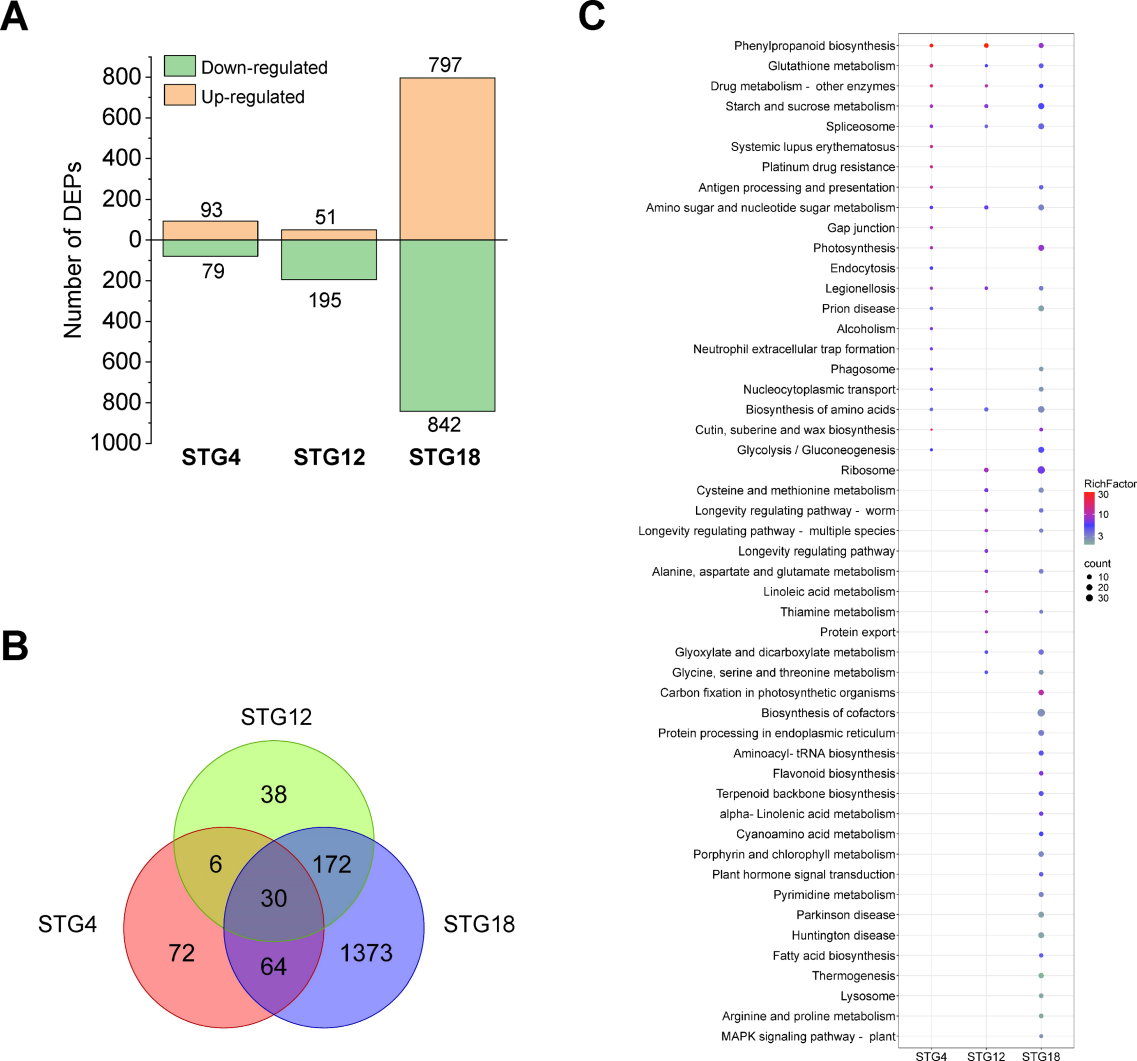
Proteome analysis under salt stress during the germination stage. **(A)** Number of differentially expressed proteins (DEPs) in QSM2 under normal and salt stress conditions at 4, 12, and 18 h. **(B)** Venn diagram of DEPs in ST under normal and salt stress conditions at 4, 12, and 18 h. **(C)** Enriched KEGG pathways for DEPs in ST under normal and salt stress conditions at 4, 12, and 18 h.

A total of 25,364 peptides together with 5,589 proteins in QSM2 were determined and differentially expressed proteins (DEPs) with a p value < 0.05 and a fold-change >1.2 or < 0.83 were identified. Compared to normal conditions, 172, 246, and 1,639 DEPs were identified in STG4S, STG12S, and STG18S, respectively (**Figure 4A**). There were 30 overlapping proteins among the three varieties and these were considered to be the essential salt-responsive proteins in triticale (**Figure 4B**). Similar expression patterns were observed for them at the three time points (**Supplementary Table S7**), suggesting they had vital functions when responding to salt stress. To achieve further insights, the DEPs were subjected to GO and KEGG enrichment analyses. Overall, STG2S, STG12S, and STG18S were obviously enriched with 82, 314, and 119 GO terms, respectively (**Supplementary Table S7**). A total of 21, 19, and 67 KEGG pathways had enriched DEPs in STG2S, STG12S, and STG18S, respectively (**Supplementary Figure S4C**; **Supplementary Table S8**). The different salt treatment time points resulted in divergent KEGG pathways (**Figure 4C**). Eight pathways: amino sugar and nucleotide sugar metabolism, starch and sucrose metabolism, drug metabolism-other enzymes, glutathione metabolism, legionellosis, phenylpropanoid biosynthesis, biosynthesis of amino acids, and spliceosome, were shared by the three comparison varieties. Notably, the phenylpropanoid biosynthesis and glutathione metabolism pathways involved in the plant stress response (Cheng et al., 2023) were significantly enriched.

### 3.5 Combined transcriptomic. proteomic and GWAS results

**Figure 5:**
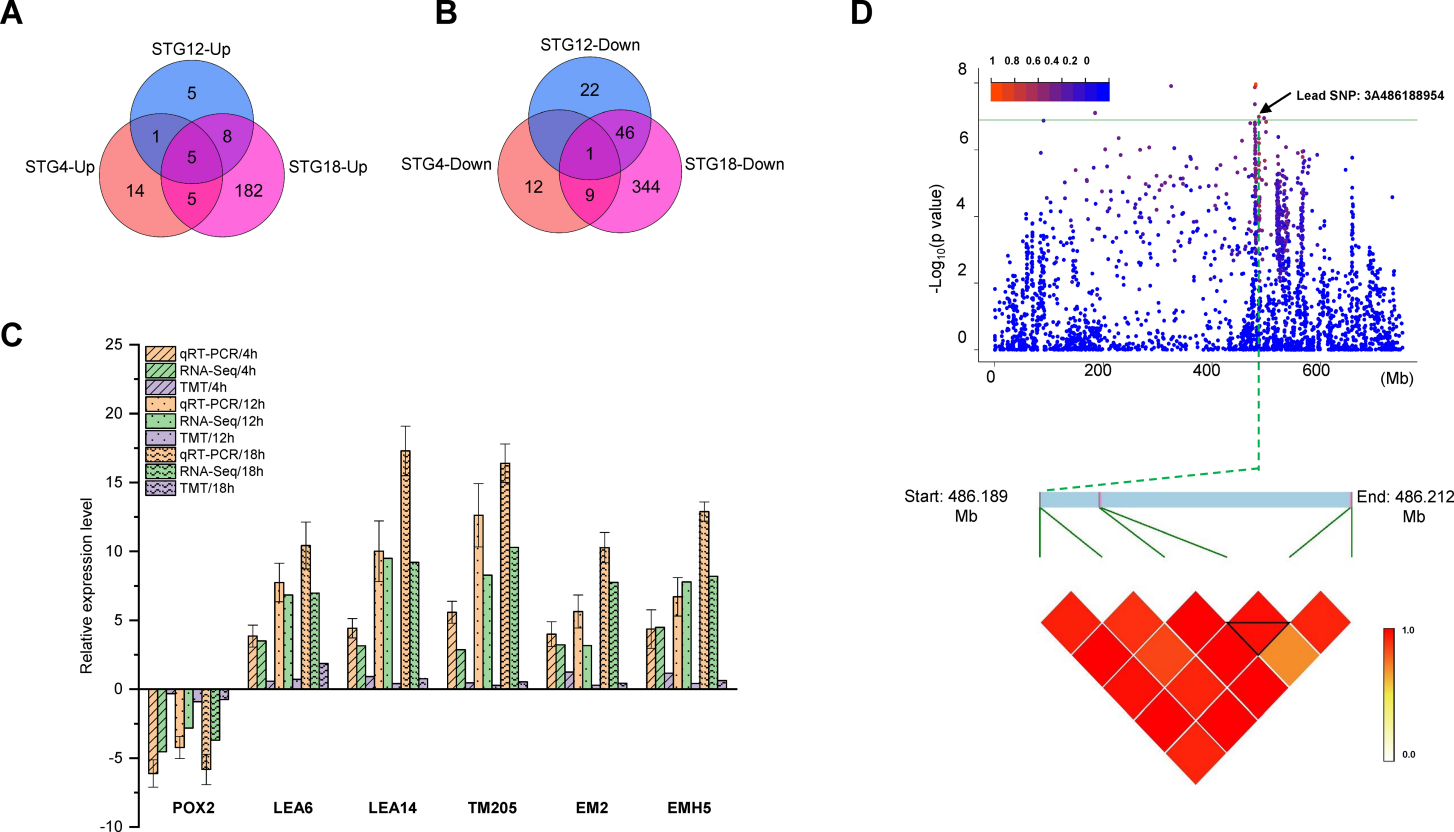
Combined transcriptomic and proteomic GWAS results. Number of up-regulated **(A)** and down-regulated **(B)** differentially expressed genes (DEGs) or proteins (DEPs) at the transcriptomic and proteomic levels in QSM2 under salt stress. **(C)** Comparison between the qRT-PCR/iTRAQ and RNA-seq results for DEGs/DEPs. **(D)** Manhattan plots for the RGR trait. Horizontal green line indicates the threshold and the points above the threshold are significant SNPs. Point indicated by the black arrow is the lead SNP in the repeatedly detected QTLs. Local Manhattan plot (top) and LD heat map (bottom) surrounding the peak on chromosome 3A.

The DEGs that were co-expressed at both, the transcriptomic and proteomic levels were selected (**Supplementary Table S9**). Our analysis demonstrated that DEGs and DEPs showed a positive correlation at 4 h (r = 0.46), 12 h (r = 0.80), and 18 h (r = 0.64) (**Supplementary Figure S2**). At 4 h, 67 genes were co-expressed at both the transcriptomic and proteomic levels, the expression trends for 47 genes were consistent at both the transcriptomic and proteomic levels, and 20 genes showed opposite expression patterns (**Supplementary Table S9**). Likewise, at the 12 h timepoint, 88 genes were co-expressed at the two levels, all of which showed a consistent expression trend (**Supplementary Table S9**). At 18 h, the number of genes co-expressed at both levels was 688, the number that were consistently expressed at each level was 600, and the number with opposite expressions was 88 (**Supplementary Table S9**). In addition, the expression trends for six genes at the transcriptomic level were consistent with those for the proteome level at all three time points and the up-regulation of five genes and down-regulation of one gene were detected (**Figures 5A, B**). A qRT-PCR was conducted to validate these six genes for their relative expression levels and their expression trends were consistent with the expression patterns measured by RNA-seq (**Figure 5C**). In addition, the common KEGG pathways that were significantly enriched after the transcriptomic or proteomic analyses were further analyzed (**Supplementary Table S10**; **Supplementary Figure S3**). A total of 16, 23, and 58 KEGG pathways were shared at 4, 12, and 18 h, respectively. The results for nine pathways: cutin, suberine, and wax biosynthesis, phenylpropanoid biosynthesis, photosynthesis, amino sugar and nucleotide sugar metabolism, cyano-amino acid metabolism, starch and sucrose metabolism, linoleic acid metabolism, spliceosome, and glutathione metabolism, were similar for all three time points (**Supplementary Table S10**).

In the GWAS results, a significant SNP, 3A486188954 (position: Chr3A-86188954), was repeatedly identified by multiple models on chromosome 3A and explained 20.15% of the phenotypic variation (**Figure 5D**). The effect of MTA on RGR was further investigated and the region was identified as an LD block (**Figure 5D**). Two genes associated with the MTA were obtained. The two genes encoded *LEA14* (TraesCS3A03G0649800) and the Germin-like protein 1-2 (TraesCS3A03G0649900). In addition, the TraesCS3A03G0649800 was up-regulated in ST (**Supplementary Table S3**). The combined transcriptomic and proteomic GWAS results showed that only one gene, late embryogenesis abundant protein (TraesCS3A03G0649800.1 and TraesCS3A03G0649800.2), showed significantly different levels in the combined transcriptomic and proteomic analysis (**Supplementary Table S3**).

The promoter region of *LEA14* contained various stress-responsive cis-acting regulatory elements, such as CAAT-box, MYB, and ABRE, as demonstrated by the promoter cis-element analysis (**Supplementary Table S11**). The *LEA14* gene in hexaploid triticale possessed relatively abundant stress-responsive elements that have been identified as promoters. This indicated that *LEA14* probably performed crucial functions in the response and tolerance to salt stress in hexaploid triticale. As the *LEA14* promoter region contained these cis-elements, conjugation with TFs might dynamically regulate *LEA14* transcription and LEA14 could take advantage of transcriptional regulation to affect salt stress in hexaploid triticale.

Overall, 2,389, 1,076, and 2,818 differentially expressed TFs were obtained in ST1, ST2, and ST3, respectively, and 170 TFs were shared by all three groups (**Supplementary Figure S4B**). Of these, 32 TFs showed an up-regulated trend and 63 TFs were down-regulated (**Supplementary Figures S4C, D**). The relative expression levels of 13 genes selected from among the 170 differentially expressed TFs were validated by qRT-PCR and the RNA-seq showed they had consistent expression patterns (**Supplementary Figure S4E**). Notably, a significant number of *ERF* (21), *NAC* (14), *MYB-related* (12), and *bHLH* (11) genes were activated following salt treatment and other TFs had 1–9 activated members (**Supplementary Figure S4A**). These TFs could bind to cis-elements in *LEA14* and regulate salt responses.

## 4 Discussion

Drought stress, salt stress, and other abiotic stresses substantially threaten current crop production. Therefore, salt-tolerant crops are needed to utilize marginal land. Hexaploid triticale is endowed with the best characteristics of wheat and rye, including a large biomass and stress resistance. Thus, elucidating the molecular mechanisms that facilitate salt tolerance has important significance for the efficient breeding of hexaploid triticale and the effective utilization of salt land. Salt tolerance is a complicated trait under the control of diversified loci. Loci and candidate genes that are responsive to salt stress have been screened from various crops at the seedling and adult stages by multi-omic approaches (Ma et al., 2021; Tu et al., 2021; Yuan et al., 2019). Other researchers focused on understanding the physiological mechanism underlying triticale responses to salt stress (Ramadan et al., 2023). Transcriptome, proteome, and GWAS analyses were performed in this study using high-quality wheat and rye genomes. We identified 81 MTAs related to 10 salt stress traits shown by hexaploid triticale at the germination stage and these explained 0.71% to 56.98% of the phenotypic variation. Of these MTAs, 31 were significantly related to more than one trait and were pleiotropic loci. The D value, which was calculated using multiple indexes and methods (including PCA, SFA, and weight), can be used in comprehensive evaluations of salt tolerance (He et al., 2022). A total of 28 MTAs related to each other by their D value were detected and 17 of these were significantly close to multiple traits, such as RGP and RGR. This information will provide a reference for the fine-mapping of salt resistance genes in hexaploid triticale.

Several candidate genes associated with MTAs were shown to take part in salt tolerance. Based on the small LD distance value for the 153 hexaploid triticale accessions (Cao et al., 2022), 54 genes related to 26 significant MTAs were obtained. According to their annotations, 13 candidate genes from 12 MTAs were likely to be linked to salt tolerance in hexaploid triticale. Of these, two candidate genes were located on chromosome 1R and the others were on chromosomes 2A, 2B, 3A, 4B, 5B, and 7B. Genes, such as *calcium-dependent kinase* from rice (Asano et al., 2012; Mahajan et al., 2008), *serine/threonine-protein phosphatase* from rice (Chen et al., 2014; Liu et al., 2000; Liao et al., 2016), *dirigent protein* from maize (Wang et al., 2022), *late embryogenesis abundant protein* from sweet potato (Jia et al., 2014; Park et al., 2011), and TF *bZIP* from wheat (Schütze et al., 2008; Zhang et al., 2008), when overexpressed, have been shown to improve salt tolerance in plants. Such genes theoretically could also enhance the salt tolerance of hexaploid triticale, but their function in hexaploid triticale needs to be confirmed. Although these candidate genes for salt tolerance have not been verified, they could provide a reference for the gene mining of salt tolerance.

To understand the expression characteristics of these candidate genes, transcriptome and proteome analyses were performed on salt-tolerant variety QSM2 at the seed germination stage when it was under salt treatment. The number of DEGs that were co-expressed at both the transcriptomic and proteomic levels showed an increasing trend under salt stress as germination time increased. This suggested that several genes or proteins responded to salt stress. Notably, six genes showed the same trend in expression at the transcriptomic and proteome levels. The responses of nine common KEGG pathways responding to salt stress: starch and sucrose metabolism, cutin, suberine, and wax biosynthesis, phenylpropanoid biosynthesis, photosynthesis, amino sugar and nucleotide sugar metabolism, cyano-amino acid metabolism, glutathione metabolism, linoleic acid metabolism, and spliceosome, were similar at all three time points. This suggested that the six genes and nine pathways played vital roles in the response to salt stress. Furthermore, some key TFs were activated following salt treatment, including *ERF*, *NAC*, *MYB-related*, and *bHLH*. These TFs could bind to the cis-elements in *LEA14* and regulate salt responses.

We performed a conjoint analysis using multi-omics. A significant and reliable MTA, 3A486188954, related to RGR, was identified on chromosome 3A and explained 20.15% of the phenotypic variation. *LEA14* (TraesCS3A03G0649800) was found to be associated with the MTA and showed up-regulated expression in ST. As corroborated by prior studies, *LEA14* overexpression can strengthen salt tolerance in sweet potato and *Arabidopsis* (Jia et al., 2014; Park et al., 2011). A promoter cis-element analysis showed that the promoter region of *LEA14* contained important stress-responsive elements (such as CAAT-box, MYB, and ABRE). These elements could bind with several TFs (including *ERF*, *NAC*, *AF2*, and *bZIP*), which further affected plants by modulating salt stress (Magwanga et al., 2018). This indicated that *LEA14* was a pivotal participant in the salt stress response shown by hexaploid triticale and might be regulated by TFs. Further experiments are needed to understand its specific function in the salt tolerance response shown by hexaploid triticale. The above results could provide candidate genes for the critical regulation of salt tolerance in hexaploid triticale.

## 5 Conclusions

In this study, the salt tolerance of 153 hexaploid triticale varieties was comprehensively evaluated and the most salt-tolerant variety, QSM2, was selected. A GWAS identified 81 MTAs associated with 10 salt-tolerance traits in the 153 hexaploid triticale accessions and these MTAs explained 0.71% to 56.98% of the phenotypic variation. A multi-omics analysis revealed that some structure genes (*LEA14*, *LEA6*, *EMH5*, *EM2*, *TM205*, and *POX*) and TFs (*ERF*, *NAC*, *MYB*, *bHLH*, *WAKY*, and *bZIP*) responded to salt stress (**Supplementary Figure S5**). Some important pathways, including the plant MAPK signaling pathway, phenylpropanoid biosynthesis, glutathione metabolism, starch and sucrose metabolism, and photosynthesis, experienced activation under salt treatment in hexaploid triticale, especially phenylpropanoid biosynthesis (**Supplementary Figure S5**). This study revealed the molecular responses to salt stress during seed germination based on multi-omics methods and the results can provide a basis for gene cloning as well as the further molecular analysis of salt stress in hexaploid triticale.

## Data Availability

The datasets presented in this study can be found in online repositories. The names of the repository/repositories and accession number(s) can be found in the article/**Supplementary Material**.

## Supplementary material

Supplementary Figure S1 Comparison of GP **(A)**, GR **(B)**, SL **(C)**, RL **(D)**, RN **(E)**, SFW **(F)**, RFW **(G)**, FW **(H)**, SDW **(I)**, RDW **(J)** and DW **(K)** under control (CK) and salt treatment. The difference between subpopulations was analyzed by t tests.

Supplementary Figure S2 The Pearson correlation coefficient (r) of expressed transcripts and proteins in ST between normal and salt stress conditions at 4h (STG4), 12h (STG12) and 18 h (STG18).

Supplementary Figure S3 The enriched common KEGG pathways for DEPs and DEGs in transcriptomic or proteomic.

Supplementary Figure S4 The statistics of differentially expressed TFs in ST between normal and salt stress conditions at 4h (STG4), 12h (STG12) and 18 h (STG18). **(A)** The types and number of differentially expressed TFs. Venn diagram of total **(B)**, up-regulated **(C)** and down-regulated **(D)** differentially expressed TFs in ST between normal and salt stress conditions at 4, 12, and 18 h. **(E)** Correlation between RNA-seq and qRT-PCR.

Supplementary Figure S5 Response mechanism of hexaploid triticale to salt stress.
