# Supplementary figures and images for: Multi-omics analysis of hexaploid triticale that show molecular responses to salt stress during seed germination
Source: Front Plant Sci. 2025 Jan 21;15:1529961. doi: 10.3389/fpls.2024.1529961 (PMC11790569; doi:10.3389/fpls.2024.1529961)

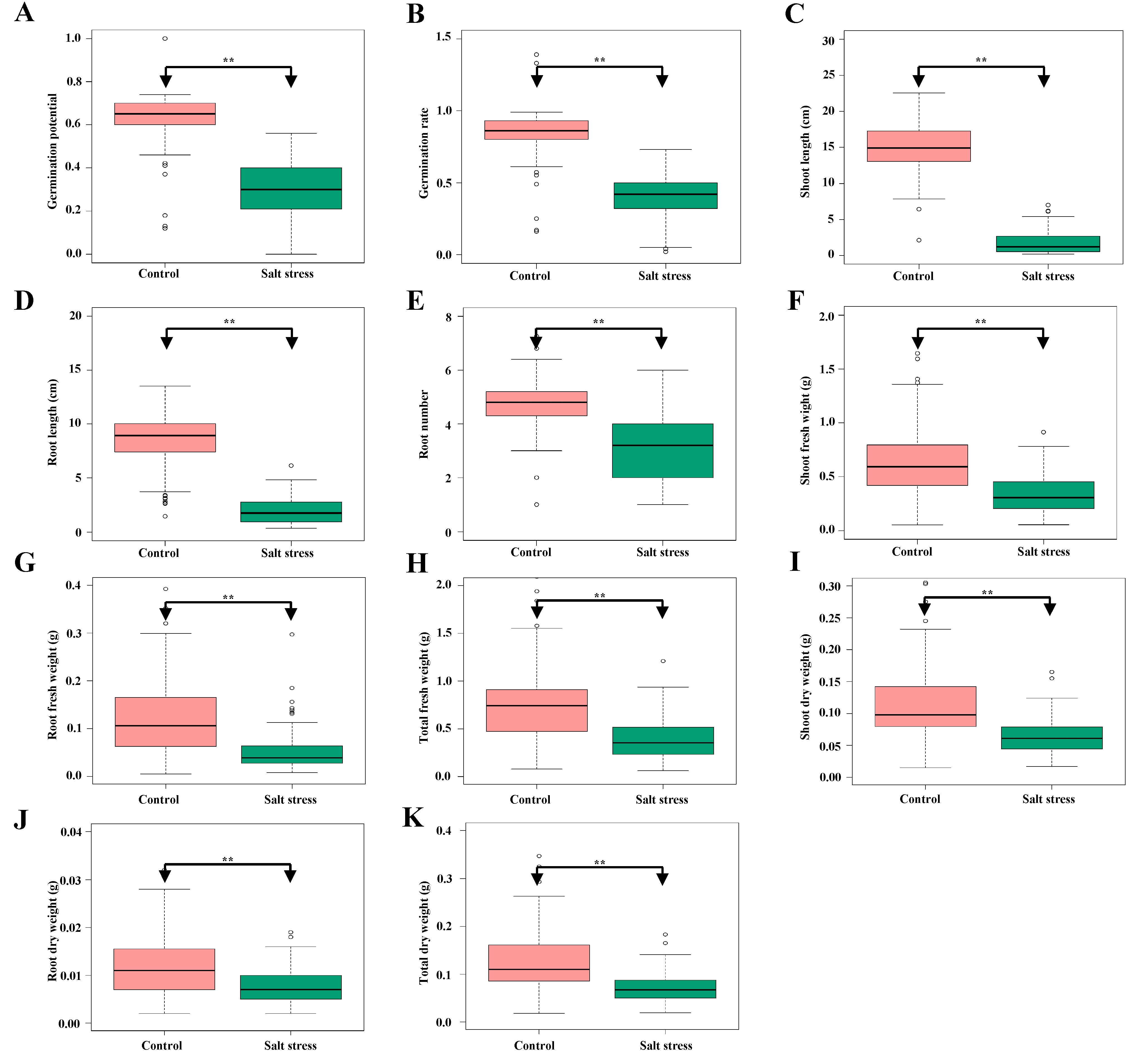

Supplement: Supplementary Figure S1 — Comparison of GP (A), GR (B), SL (C), RL (D), RN (E), SFW (F), RFW (G), FW (H), SDW (I), RDW (J) and DW (K) under control (CK) and salt treatment. The difference between subpopulations was analyzed by t tests. [file Image1.tiff]

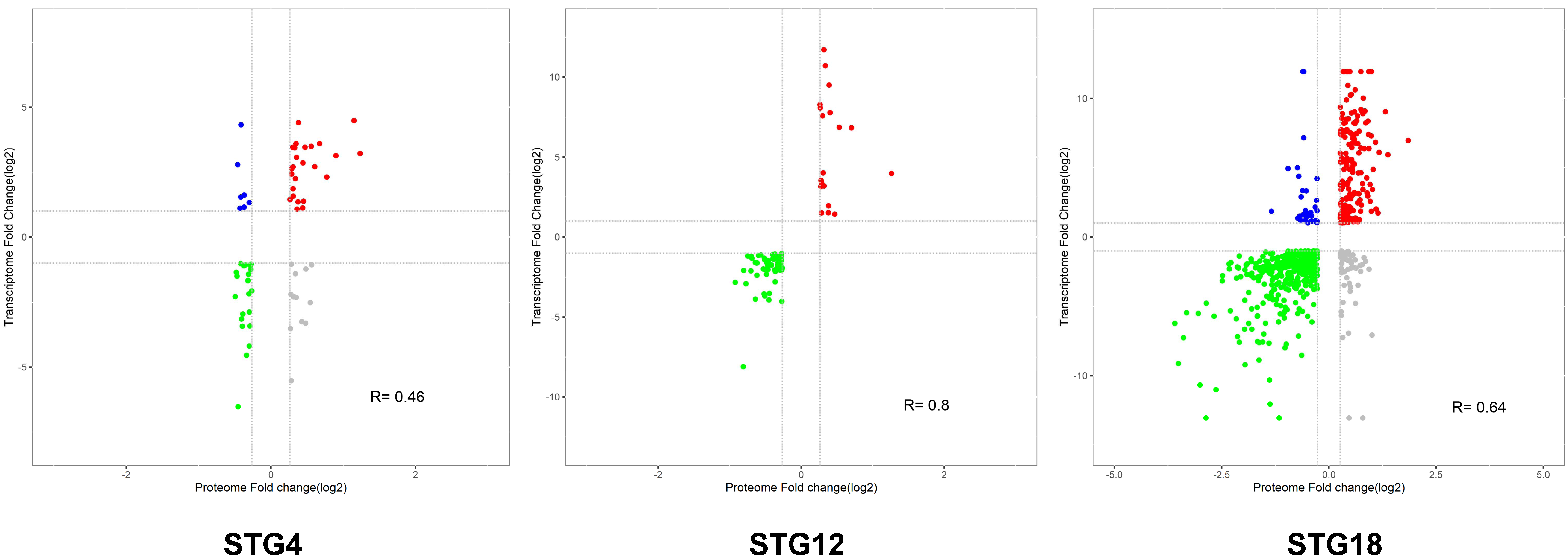

Supplement: Supplementary Figure S2 — The Pearson correlation coefficient (r) of expressed transcripts and proteins in ST between normal and salt stress conditions at 4h (STG4), 12h (STG12) and 18 h (STG18). [file Image2.tiff]

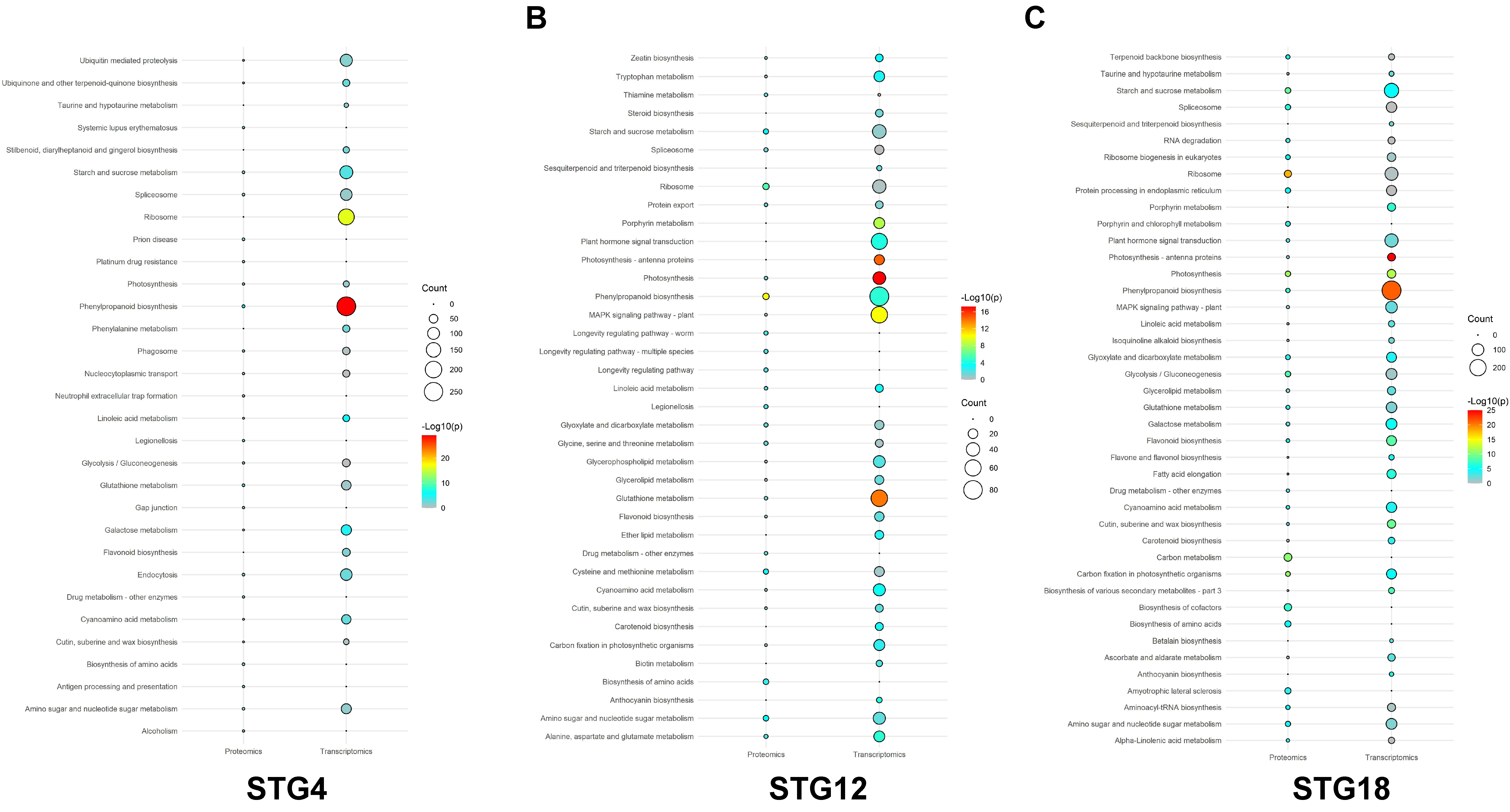

Supplement: Supplementary Figure S3 — The enriched common KEGG pathways for DEPs and DEGs in transcriptomic or proteomic. [file Image3.tiff]

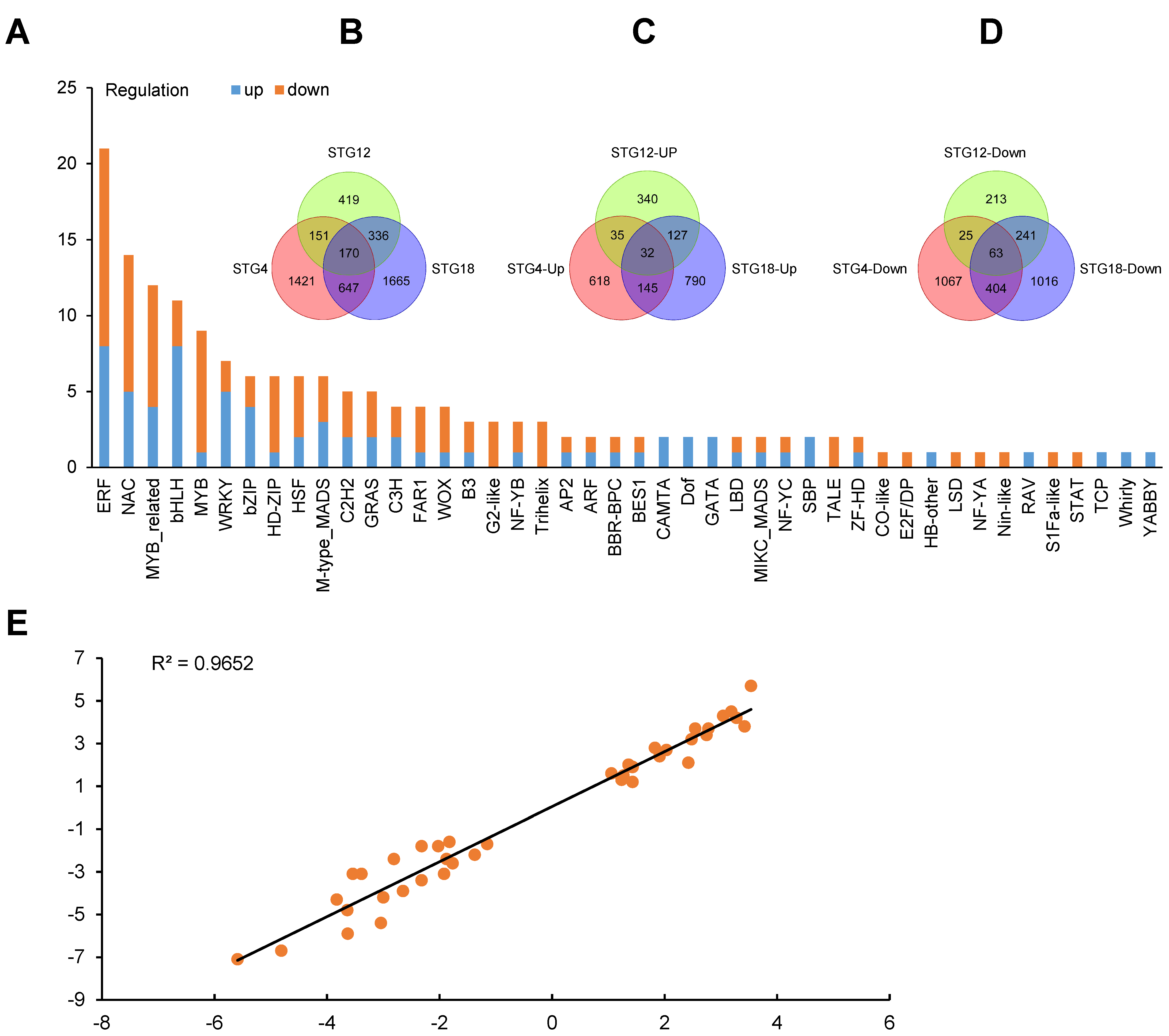

Supplement: Supplementary Figure S4 — The statistics of differentially expressed TFs in ST between normal and salt stress conditions at 4h (STG4), 12h (STG12) and 18 h (STG18). (A) The types and number of differentially expressed TFs. Venn diagram of total (B), up-regulated (C) and down-regulated (D) differentially expressed TFs in ST between normal and salt stress conditions at 4, 12, and 18 h. (E) Correlation between RNA-seq and qRT-PCR. [file Image4.tiff]

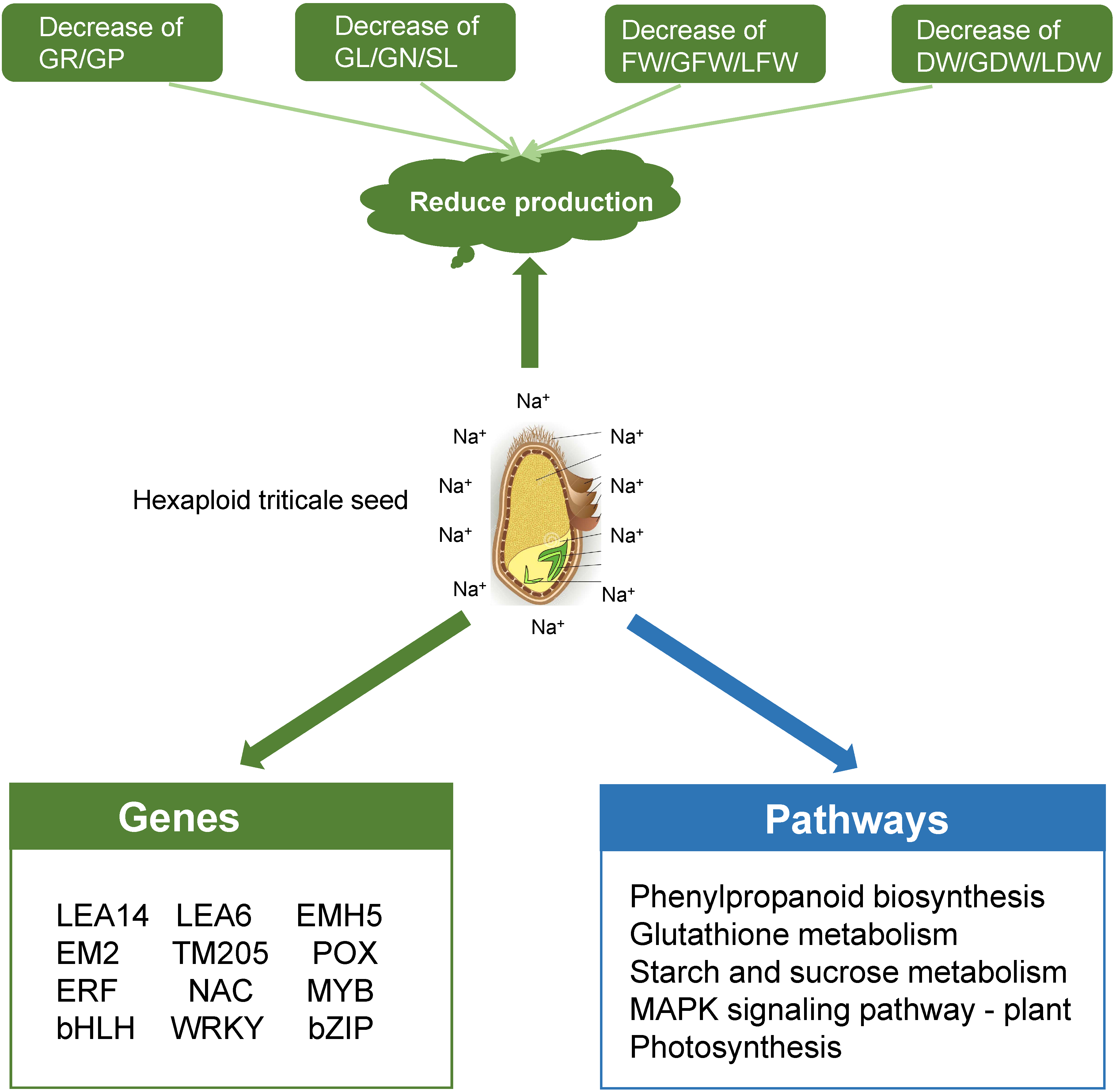

Supplement: Supplementary Figure S5 — Response mechanism of hexaploid triticale to salt stress. [file Image5.tiff]
